# Homofermentative *Lactobacilli* isolated from organic sources exhibit potential ability of lactic acid production

**DOI:** 10.3389/fmicb.2023.1297036

**Published:** 2023-12-21

**Authors:** Jain Maria Stephen, Arabi Mohammed Saleh

**Affiliations:** ^1^School of Bio Sciences and Technology, Vellore Institute of Technology, Vellore, India; ^2^VIT School of Agricultural Innovations and Advanced Learning, Vellore Institute of Technology, Vellore, India

**Keywords:** lactic acid, lactic acid bacteria, homofermentative, organic sources, *Lactobacilli*

## Abstract

There has been an increasing interest in recent years in lactic acid bacteria that are derived from organic sources for lactic acid production. This research article presents the isolation and identification of homofermentative lactic acid bacteria from various novel organic sources, followed by qualitative and quantitative analyses of lactic acid produced. A total of 32 isolates were identified initially from various sources, such as curd (C1, C2), probiotics (P1, P2, and P3), silage (Si1 and Si2), soil samples (S1, S2, and S3), vermicompost (V1 and V2), and Farmyard manure. Biochemical tests such as Gram’s staining, catalase test, and oxidase test were conducted for preliminary identification of lactic acid bacteria using De Man, Rogosa, and Sharpe agar (MRS) media. Through selection and identification, based on colony morphology and biochemical characteristics, 18 isolates were identified as lactic acid bacteria. The subsequent analysis included a tube test, screening for organic acid production, and homofermentative screening using homofermentative–heterofermentative differential (HHD) medium for qualitative analysis of lactic acid. The results revealed that 9 out of 18 selected strains were homofermentative and had promising potential for the production of lactic acid. Furthermore, six isolates (P1-1, S1-3, C2-1, V2-3, P2-1, and C1-1) from all of the nine positive strains were subjected to pH testing (0, 24, 48, and 72 h) and titrimetric assay for estimation of % crude lactic acid present. The presence of lactic acid was confirmed using thin-layer chromatography (TLC). L (+)-Lactic acid was quantified using a K-LATE enzymatic assay kit, for the best three isolates (P1-1, S1-3, C2-1). Finally, the strains were subjected to 16SrRNA sequencing and were identified as *Lactobacilli*. Based on the findings of the study, it could be concluded that homofermentative lactic acid bacteria with significant LA-producing ability can be obtained from different organic sources and may prove to be useful in the successful production of lactic acid for biotechnological applications.

## 1 Introduction

The investigation of lactic acid (LA) production dates back to the year 1780, while its commercial application was established in 1881, marking its progression as an advanced technology (Zwiercheczewski de Oliveira et al., 2022). LA, an essential organic acid, possesses a diverse array of applications across various domains encompassing the preservation of human food products and other industrial utilization (Rodrigues et al., 2017). It is chemically referred to as 2-hydroxypropanoic acid which is the most commonly found hydroxycarboxylic acid. In the food and non-food industries, including the pharmaceutical and cosmetic sectors, the use of natural organic acids, particularly LA, has been well-documented for many years. These applications include the synthesis of oxygenated compounds, plant growth promoters, and special chemical reagents (Sobrun et al., 2012). Nowadays, there is a greater need for LA as a feedstock for the production of biopolymer poly-lactic acid (PLA), which is an environmentally benign, biocompatible, and biodegradable alternative to plastics derived from petrochemicals. PLA has the potential to replace traditional polymers such as polycarbonate (PC), polystyrene (PS), and polyethylene terephthalate (PET) for packaging in cars and electronic devices (Nair et al., 2016). LA is additionally regarded as a platform chemical owing to its ease of conversion into a number of industrially vital chemicals, such as 2,3-pentanedione, acrylic acid, lactate esters, propylene oxide, and propylene glycol (Datta et al., 1995). Although the world’s current estimate of its ability to produce LA is 150,000 metric tons annually, the market for LA derivatives is anticipated to grow considerably in the near future (Moon et al., 2012). Finding innovative and affordable sources for effective production is crucial since LA and products based on LA are expected to find more applications in the coming years. While bacterial fermentation primarily utilizes simple sugars and is a key method in biotechnological manufacturing, it also accounts for more than half of the current total acid capacity (Huang et al., 2003). The production and effectiveness of LA synthesis are influenced by various factors, including the pH level, temperature, and presence of essential nutrients like amino acids, nucleotides, peptides, and vitamins. The selection of lactic acid bacterial (LAB) strains is also a contributing factor (Abedi and Hashemi, 2020). Organic molecules act as both donors and acceptors of electrons during the energy-producing process of fermentation. When a molecule undergoes metabolism, it loses some of the energy that it previously collected. As a result, LAB are frequently employed as an inexpensive means of preserving food through fermentation, which typically requires very little or no heat (Boontawan et al., 2011). In addition, the process of fermentation is highly efficient and cost-effective as it operates without the need for complex procedures. Furthermore, there is a reduction in energy consumption and a decrease in the cost of raw materials. According to Abedi and Hashemi, the utilization of renewable sources and the employment of microorganisms (Abedi and Hashemi, 2020) capable of producing a sole isomer present a potential advantage in comparison with chemical synthesis (Bernardo et al., 2013). LA has been synthesized by various species such as *Rhizopus* fungi and diverse genera of bacteria, especially LAB. Numerous bacteria, such as *Enterococcus, Carnobacterium, Lactococcus, Lactobacillus, Oenococcus, Leuconostoc, Tetragenococcus, Pediococcus, Clostridium, Vagococcus*, and *Weissella*, have been found to produce LA. These sources for the production of LA have been extensively studied (Huang et al., 2003). *Rhizopus* sp. can manufacture L (+) lactic acid from starch, but the yield is very low as compared to LAB (Ghaffar et al., 2014). Optically pure LA can be effectively synthesized by commercial large-scale microbial fermentations without the need for downstream processing to separate the two enantiomers, namely, L(+)-lactic acid and D(−)-lactic acid (Campos et al., 2023).

LAB are a group of bacteria that are Gram-positive, non-spore-forming, and lacking motility. This group encompasses various species that possess the capability to metabolize carbohydrates and generate LA as their principal final product. LAB are prominently utilized in the production of LA which typically exhibit a cocci or rod-shaped morphology and possess a notable capacity to withstand acidic conditions. These bacteria can grow in temperatures ranging from 5 to 45°C and in a pH range of 3.5–10.0; therefore, the ideal conditions for growth vary depending on the producers. According to Mohanty et al., these bacteria need certain substances to flourish, including carbon and nitrogen sources such as amino acids, carbohydrates, vitamins, and minerals (Mohanty et al., 2015). LAB encompass an extensive variety of over 60 genera with *Lactobacillus* species being particularly notable for their intriguing fermentation capabilities (Wang et al., 2021). LAB can be divided into two physiological groups—heterofermentative and hоmоfеrmеntative, based on their hexose fermentation pathways (Zúñiga et al., 1993). Heterofermentative LAB use the pentose-phosphate pathway and produce LA, along with other by-products such as carbon dioxide, acetic acid, and ethanol. Homofermentative LAB convert hexoses to LA mainly through the glycolysis process, with aldolase enzyme allowing for almost complete glucose conversion (Narvhus and Axelsson, 2003). They can also utilize both hexose and pentose carbohydrates through the Embden–Meyerhof pathway. The primary by-product of homofermentative LAB is two LA molecules per mole of glucose ingested; the theoretical yield is 1 g.g^−1^, and experimental yields vary depending on the kind of carbon source utilized (Castillo Martinez et al., 2013). Furthermore, homofermentative LAB have been proposed as highly favorable microorganisms for industrial applications owing to their significant commercial viability in areas such as food and bio-preservations, probiotic utilization, and biodegradation procedures. Therefore, the identification of a homofermentative LAB is a critical factor in developing an economical bioprocess for LA production. The major homofermentative candidates that can be used in the production of LA include the genera *Lactococcus*, *Lactobacillus*, and *Enterococcus* (Moon et al., 2012). Bacteria such as *Lb. casei, Lb. plantarum, Lb. delbrueckii, Lb. rhamnosus,* and others use substrates with a high concentration of sugars, including agro-industrial wastes, to produce high LA titers, especially, L(+)-lactic acid enantiomer (>95%), owing to their selective metabolism (Zwiercheczewski de Oliveira et al., 2022). After polymerization, high-purity L (+) lactic acid can be utilized as an alternative source for commercial plastic by forming crystalline PLA. PLA provides a further environmental benefit compared to petrochemical-derived plastics due to its lack of net carbon dioxide emissions into the atmosphere (Sangeetha et al., 2018). In recent years, the production of LA by LAB strains sourced from various organic materials has become increasingly important in the field of biotechnology. Microorganisms that are utilized in commercial applications exhibit a rapid fermentation rate when utilizing low-cost raw materials, require minimal amounts of nitrogen-based nutrients, produce minimal quantities of cell biomass and other by-products in small quantities, and yield high concentrations of the desired stereo-specific LA under high temperatures and low pH conditions (Narayanan et al., 2004). The most economically beneficial strains within the LAB group are *Lactobacillus* strains due to their high productivity, high yield, and high acid tolerance. It is possible to alter them in a controlled manner to generate specifically L/D-lactic acid (Kylä-Nikkilä et al., 2000). The food and pharmaceutical sectors are currently concentrating heavily on obtaining new strains of probiotic bacteria from rare and unique conventional sources that have adequate practical, technological, and therapeutic potential. In recent years, probiotics have been developed using lactic acid bacterial species such as *Lb. rhamnosus, Lb. casei*, and *Lb. johnsonii* that have been recognized for immense health benefits (Shirisha et al., 2021). The disadvantages of using LAB for commercial LA production include high nutrient demands and contamination risks due to low fermentation temperatures (Abedi and Hashemi, 2020). Due to its high yield (almost at the maximum theoretical value), productivity, and excellent optical purity (>99%) of LA, homofermentative LAB are the sole method available for commercial production (more than 100 g/L of lactic acid) of LA (Abedi and Hashemi, 2020). According to Abdel-Rahman et al., engineered *Lb. plantarum* and *Enterococcus mundtii* QU 25 could also metabolize pentose sugars to LA (Abdel-Rahman et al., 2013) homofermentatively. Due to the frequent presence of LAB in our ecological surroundings, it is imperative to isolate this microorganism for the purpose of identifying its numerous benefits (Ekundayo, 2014). Investigating LAB found in unidentified niches could result in the isolation of rare species. They are acclimated to acidic conditions and high sugar concentrations, although their classification is rather complicated. Manipulating the genes of LAB and employing metabolic engineering can create resilient cell factories that are specifically designed to produce valuable products for industry and agriculture (Raman et al., 2022). Considering the prevalence of LAB in various ecosystems, our study aimed to investigate the isolation of wild LAB strains from unconventional and underexplored sources of organic origin. Current literature provides limited data on the isolation of homofermentative *Lactobacillus* strains from sources such as vermicompost and soil with high organic matter content (sugarcane soil, fish, and meat market soil). The utilization of widely available sources such as curd, commercial probiotics, and silage was also taken into account in our analysis. This study ascertains the presence of homofermentative LAB in various organic sources, with the inherent capability to facilitate significant-scale LA production for economically viable fermentation processes. In subsequent investigations, comparative analyses were conducted to determine whether the LA biosynthesis ability of the subject strains surpasses that of previously reported and commonly used LAB strains in industrial settings. This study reports a total of six homofermentative *Lactobacillus* strains that have the potential ability to produce LA, with certain strains demonstrating a higher rate of production compared to those typically found in common sources. Therefore, in terms of natural LAB strains found in the literature, our results offer an indisputable uniqueness. Further investigation, including genetic modifications, can improve the desirable qualities of these naturally occurring isolates, making them more suitable for economical and environmentally sustainable large-scale fermentation and other industrial processes.

## 2 Materials and methods

### 2.1 Sample collection and isolation of LAB from selected sources

A total of six different sample categories were chosen for the study based on an extensive literature survey and local availability. The specified information is listed as follows: 1. curd (C1—Home-made curd from different localities of Katpadi, TN, India, commercial curd samples: C2—Hatson, C3—Arogya), 2. commercial probiotics (P1—Yakult, P2—Yoghurt-Milky Mist, P3—Probiotic Tablets-Astra Labs), 3. silage (Si1—Livestock Research Station, Kattupakkam, TN, India, Si2—Cornext Agri Products, Kochi, Kerala, India), 4. soil (S1—Vellore Main Market-Slaughter house, S2—Vellore Main Market-Fish market, Vellore, TN, India, S3—Sugarcane soil, Sugarcane fields, Brahmapuram, TN, India, S4—Garden soil, Premises of VIT University, Vellore, TN, India), 5. vermicompost (V1—Dept. of VAIAL, VIT University, Vellore, TN, India, V2—Sakthi Vermicompost, Vellore, TN, India), 6. farmyard manure (F1—Dept. of VAIAL, VIT University, Vellore, TN, India). All the soil samples were collected from approximately 5 cm depth from the top soil layer. For soil samples, 1 g each was weighed and placed into 9 mL of sterile distilled water (Ekundayo, 2014). Then, 7-fold serial dilutions were made from this mixed solution, and 0.1 mL from 10^−4^, 10^−5^, and 10^−6^ dilutions were spread plated on de Man, Rogosa, and Sharpe (MRS) agar plates in triplicates. For all other samples, 10 g of each sample was homogenized in 90 mL of sterile distilled water and serially diluted, 7-fold. 0.1 mL from sample suspensions (10^−4^, 10^−5^, and 10^−6^ dilutions) was spread on MRS agar plates, in triplicates (Bansal et al., 2013). The plates were incubated at 37°C for 24–48 h, except for silage samples, which were incubated at 30°C for the same time period.

Distinct colonies were selected randomly and were re-streaked on MRS agar plates and incubated at 37°C for 24 h to obtain well-isolated colonies (Goyal, 2012). These isolates were sub-cultured in MRS broth every 15 days and were stored as 50% glycerol stock solution at −80°C for long-term storage.

### 2.2 Primary characterization of the isolates

All separate colonies obtained after spread plating were then transferred to fresh MRS plates and incubated at 37°C and 30°C for 24 h for isolates from other samples and silage samples, respectively. Preliminary identification of these isolates was based on their phenotypic and biochemical characteristics that included Gram’s reaction, study of colony morphology, catalase test, and oxidase test (Garcia et al., 2016).

### 2.3 Qualitative analysis of lactic acid production

#### 2.3.1 Tube test

MRS broth supplemented with 0.04% (w/v) of bromocresol purple was prepared, and the selected strains were inoculated and then incubated in a static condition at 37°C for 48 h. The isolates showing growth and yellow color were assumed to be LAB with LA-producing capability (Klongklaew et al., 2021).

#### 2.3.2 Screening for organic acid production

Selected isolates were patched in the center (diameter∼ 0.8 cm) of MRS agar plates incorporated with 1% calcium carbonate. The plates (triplicates) were subsequently incubated at a temperature of 37°C, and the clear zone surrounding the inoculum was monitored from days 1 to 7 (Putra et al., 2018; Amarantini et al., 2019).

#### 2.3.3 Screening for homofermentative LAB

Selected isolates were grown on LAB differential agar, also called HHD media (homofermentative–heterofermentative differential media, HiMedia), for screening of homofermentative strains. 35.56 g/L of media was prepared, and 1 g of polysorbate 80 was added and boiled to dissolve. The media were poured to plates after sterilization, and isolates were inoculated. Plates were observed for change of color of the media, after 24–48 h (Zúñiga et al., 1993). HHD media is used for the differentiation of homofermentative *Lactobacilli* and heterofermentative *Streptococci.* The components of media, such as Soy peptone, Acicase™, and yeast extract, provide nitrogen and carbon-based molecules, long-chain amino acids, vitamins, and essential nutrients that promote the growth of lactic bacteria. Fructose serves as the fermentable carbohydrate of the medium. The pH indicator is bromocresol green (McDonald et al., 1987).

### 2.4 Quantitative analysis of LA production

The best six isolates based on the results “from previous experiments” were subjected to further quantitative tests. The selected strains were P1-1, S1-3, C2-1, V2-3, P2-1, and C1-1.

#### 2.4.1 pH Test

1 ml of the selected isolates was inoculated to 10 mL of MRS broth and was incubated at 37°C under shaking conditions (135 rpm). The pH values at 0 h, 24 h, 48 h, and 72 h were recorded using a digital pH meter to test the acidifying power of the microorganisms selected (Venus and Richter, 2006), and the total change in pH was also calculated (∆pH = Initial pH – Final pH).

#### 2.4.2 Titrimetric assay of LA

The titrimetric assay takes into account the titratable acidity (TA) of lactic acid present in the sample by determining the concentration of un-disassociated hydrogen ions and that of disassociated hydrogen molecules. Since the acidity of a solution is related to the amount of hydrogen present in it, TA can be a better indication of total lactic acid levels present in a given sample (Mohanty et al., 2015). Then, 0.5 mL of each culture was inoculated to 10 mL MRS broth and incubated at 37°C for 24 h at 135 rpm. These cultures were then centrifuged (8,000 rpm, 4 min) to collect the supernatants. A total of 1 mL of each of the six samples (P1-1, S1-3, C2-1, V2-3, P2-1, C1-1) were diluted in distilled water with two to three drops of phenolphthalein indicator (dilution factor 1:10) and titrated against 0.1 N NaOH until the color of the sample turns pale pink (Sobrun et al., 2012). Titration was repeated until one set of concordant values was obtained. The percentage of lactic acid was calculated using Equation 1:


(1) $$R=\frac{\left(V\times C\times Mw\times DF\times 100\right)}{1000\times Wt}$$

where R is the % of lactic acid, V is the titer value (in ml), C is the concentration of the titrant (NaOH), Mw is the molecular weight of lactic acid (90.08), D.F is the dilution factor (1/10), and Wt. is the amount of sample in ml or gram.

### 2.5 Thin-layer chromatography

The presence of lactic acid in the samples was confirmed using thin-layer chromatography (TLC). The samples were run on a TLC plate (Aluminum sheet coated with silica gel) using a 1:1 mixture of ethanol and chloroform as solvent. The plates were dried later and observed under a UV-trans illuminator for spot formation. Rf values of the standard and samples were calculated and compared. L-Lactic acid (Extrapure AR, 88%, SRL) was used as standard. Rf values were calculated for the selected strains (P1-1, S1-3, C2-1) using the standard formula: Distance traveled by solute to distance traveled by solvent.

### 2.6 Estimation of L (+)-lactic acid

Using the K-LATE 06/18 enzymatic test kit (Megazyme, Wicklow, Ireland), the amount of L(+)-lactic acid generated was measured in accordance with the manufacturer’s instructions (Bravo et al., 2017). Fresh cultures of each of the strains P1-1, S1-3, and C2-1 were prepared by inoculating 0.5 mL of inoculum in 10 mL MRS broth. Cultures were then inoculated at 37°C for 24 h at 135 rpm. The supernatants were collected after centrifugation at 10,000 rpm for 3 min. A microplate assay procedure was carried out, and the estimation of L (+)-lactic acid was done using Mega-Calc™ Data Calculator, available from the Megazyme website. The experiment was done in triplicates, and the average values were taken. This assay utilizes two enzymatic reactions. The initial reaction is facilitated by L-lactate dehydrogenase (L-LDH), an enzyme that converts L-Lactate (L-LA) to pyruvate through the oxidation process utilizing nicotinamide-adenine dinucleotide. In the second reaction, the D-GPT enzyme converts pyruvate to 2-oxoglutarate and D-alanine, with an excess of D-glutamate present. The resulting amount of NADH generated through these two coupled reactions is proportional to the amount of L (+)-lactic acid present. The amount of NADH is determined by measuring the increase in absorbance at 340 nm.

### 2.7 DNA isolation, sequencing, and blast identification

The extraction of genomic DNA from bacteria was done using the Xploregen Universal gDNA Extraction Kit (Xploregen Discoveries, India). The ~1.5 kbp, 16 s-rDNA fragment was amplified using high-fidelity PCR polymerase, in the thermal cycler BIO-RAD T100 using forward and reverse primers, which were (Sequence 5’à 3`) 16 s Forward- GGATGAGCCCGCGGCCTA (72.22% GC content) and 16 s Reverse-CGGTGTGTACAAGGCCCGG (68.42% GC content), respectively, and the PCR product was sequenced bi-directionally.

In total, 143 ng of extracted DNA was used for amplification along with 10 pM of each primer. The TAQ Master mix consisted of High-Fidelity DNA Polymerase, 0.5 mM dNTPs, 3.2 mM MgCl₂, and PCR enzyme buffer. The optimum cycling conditions for PCR amplification involved an initial denaturation step for 3 min at 94°C followed by 30 cycles of denaturation for 1 min at 94°C, annealing for 1 min at 50°C, extension for 2 min at 72°C, and final extension for 7 min at 72°C. The PCR amplification conditions were DNA—1 μL, 16 s forward primer—2 μL, 16 s reverse primer—2 μL, dNTPs (2.5 mM each)—4 μL, 10X Taq DNA polymerase assay buffer—10 μL, Taq DNA polymerase enzyme (3 U/ ml)—1 μL, and water-30 μL, and the total reaction volume was 50 μL. The PCR products were confirmed on agarose gel (1.2%) electrophoresis. The PCR products were then sequenced, and the sequence data were checked for similarity analysis with NCBI, BLAST (basic local alignment search tool) program and submitted to the GenBank sequence database for accession numbers (Moon et al., 2012). 16 s molecular identification (MID) and phylogenetic analysis for the strains (P1-1, S1-3, C2-1, V2-3, P2-1, C1-1) were performed by Biokart India Pvt. Ltd., Bengaluru.

### 2.8 Statistical analysis

All the experiments were performed in triplicates for comparison and determination in screening and characterization. The results for quantitative analysis are presented as means ± standard deviation. All the calculations were performed using the software JMP Pro version 17, NC.

## 3 Results

### 3.1 Isolation of LAB

LAB were obtained from various sources and cultured on MRS agar, a selective medium for LAB that facilitates their robust growth while inhibiting the growth of other bacterial species. Initially, a total of 32 isolates were isolated from the six selected samples. A single isolate was acquired from C1 and subsequently named as C1-1. Two isolates derived from C2 were designated as C2-1 and C2-2, respectively. From C3, three isolates were derived and named as C3-1, C3-2, and C3-3. Three strains were obtained from each of the three commercial probiotics, and they were designated as P1-1, P2-1, and P3-1, corresponding to the strains obtained from P1, P2, and P3, respectively. A total of six isolates were acquired from Si1, denoted as Si1-1, Si1-2, Si1-3, Si1-4, Si1-5, and Si1-6, while the isolates obtained from Si2 were designated as Si2-1 and Si2-2. The isolates derived from three soil samples were designated as S1-1, S1-2, S1-3, S1-4, and S1-5 for sample S1, S2-1 and S2-2 for sample S2, and S3-1, S3-2, S3-3, S3-4, and S3-5 for sample S3. Three isolates were acquired from V2 and designated as V2-1, V2-2, and V2-3. No isolates were obtained from either sample V1 or F1.

### 3.2 Identification of colony morphology

**Figure 1:**
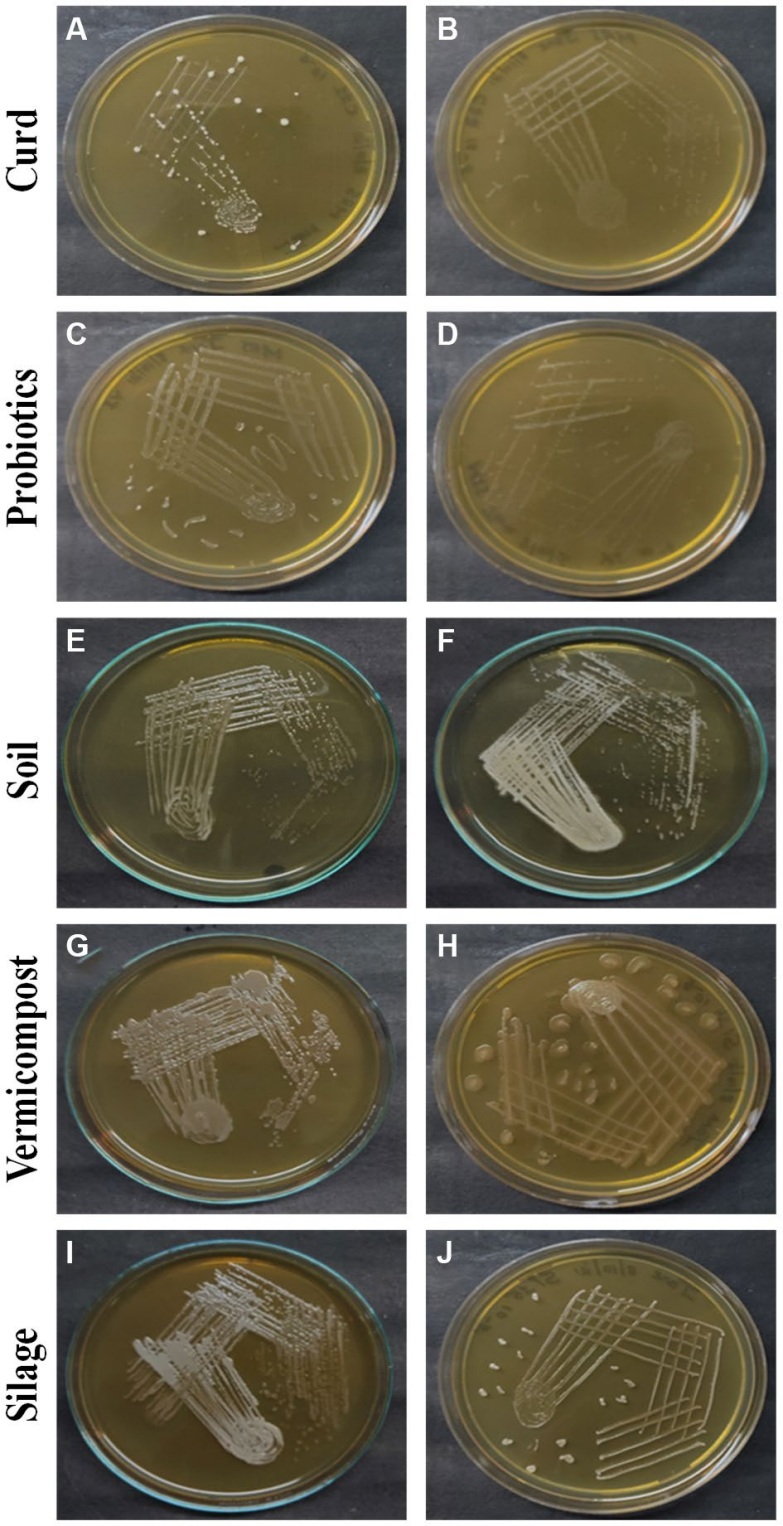
LAB colonies that develop on the MRS medium from different sources: curd **(A)** C1-1, **(B)** C2-2, probiotics **(C)** P1-1, **(D)** P2-1, soil **(E)** S1-3, **(F)** S3-4, vermicompost **(G)** V2-2, **(H)** V2-3, and silage **(I)** Si1-4, **(J)** Si1-5.

Figure 1 illustrates the observable growth of the isolates on MRS agar. Distinct colonies with varying characteristics were observed following a 24-h incubation period, indicating successful isolation. Based on observations made on the colonies that developed on the MRS medium by streak plate, it was determined that the colonies of LAB exhibited variations in their morphology, shape, color, elevation, margin, and other notable features, and the characteristic features are tabulated in Table 1.

**Table 1:** Isolated colony morphology characteristics of all the 32 isolates.

Sample	Strain	Color	Size	Shape	Elevation	Margin	Centre	Others
C1	C1-1	Creamy white	Small	Round	Flat	Entire	–	Opaque
C2	C2-1	Dull yellow, shiny	Minute	Round	Flat	Entire	–	Slightly transparent
	C2-2	Dull yellow, shiny	Minute	Round	Flat	Entire	–	Slightly transparent
C3	C3-1	White	Small	Round	Raised	Entire	–	Opaque
	C3-2	Creamy yellow	Minute	Round	Raised	Entire	–	Shiny
	C3-3	White	Small	Round	Flat	Entire	–	Slimy, slightly transparent
P1	P1-1	Dull yellow, shiny	Minute	Round	Flat	–	–	Slightly transparent
P2	P2-1	Dull white	Minute	Round	Flat	Entire	–	Slightly transparent
P3	P3-1	Creamy white	Minute	Round	Slightly raised	Entire	–	Slightly transparent
Si1	Si1-1	White	Large	–	–	Irregular	White	–
	Si1-2	Bright white	Small	Round	Raised	Entire	–	–
	Si1-3	Yellowish white	Small	Round	Raised	Entire	–	–
	Si1-4	Dull yellow	Medium	Round	Flat	Entire	–	Transparent
	Si1-5	White	Small	Round	Raised	Entire	–	–
	Si1-6	Yellowish white	Small	Round	Flat	Entire	–	Shiny
Si2	Si2-1	White	Small	Round	Raised	Entire	–	Opaque
	Si2-2	Creamy white	Minute	Round	Flat	Entire	–	Slightly transparent
S1	S1-1	Dull white	Medium	Irregular	Raised	Irregular	Thick	–
	S1-2	Dull white	Small	Round	Raised	Entire	–	Opaque
	S1-3	Creamy white	Small	Round	Flat	Entire	–	Slightly transparent
	S1-4	White	Medium	Round	Raised	Entire	Thick	Opaque
	S1-5	White	Medium	Round	Raised	Entire	–	Opaque
S2	S2-1	Dull white	Small	Round	Raised	Entire	–	Slightly transparent
	S2-2	Creamy white	Medium	Irregular	Raised	–	–	Shiny
S3	S3-1	Dull white	Minute	Round	Raised	Entire	–	Opaque
	S3-2	White	Minute	Round	Raised	Entire	White	Opaque
	S3-3	Dull	Small	Round	Raised	Irregular	Opaque	Shiny
	S3-4	White	Small	Round	Flat	Entire	–	Slightly transparent
	S3-5	Dull white	Minute	Round	Flat	Entire	–	–
V2	V2-1	White	Large	Irregular	–	Irregular	–	–
	V2-2	Dull creamy white	Minute	Oval	Flat	Raised	–	Shiny
	V2-3	Creamy white	Small	Round	Flat	Entire	–	Slightly transparent

The coloration of isolated colonies of LAB obtained from different sources exhibits a variety of hues including white, bright white, dull white, creamy yellow, or creamy white. The colonies exhibit a range of sizes, including small, medium, and large. The majority of the colonies display a round shape, with the exception of S1-1, S2-2, V2-1, and V2-2.

### 3.3 Biochemical characterization

Biochemical assays including Gram’s staining, catalase testing, and oxidase testing were conducted to identify the LAB among all 32 isolates. LAB can be distinguished through the utilization of these biochemical tests. This group of bacteria is characterized by their Gram-positive nature, as well as their morphology, which may appear as either cocci or rods. Additionally, LAB are known to be catalase-negative and oxidase-negative (Mokoena, 2017). The results of biochemical tests are given in Table 2. The findings indicate that out of the 32 isolates examined, 18 isolates exhibited characteristics consistent with Gram’s positive, catalase-negative, and oxidase-negative properties. The isolates that exhibited favorable findings for LAB include C1-1, C2-1, C2-2, P1-1, P2-1, Si1-2, Si1-3, Si1-5, S1-2, S1-3, S1-4, S1-5, S2-1, S3-1, S3-2, S3-3, V2-2, and V2-3. The aforementioned isolates were employed for subsequent investigations.

**Table 2:** Biochemical characteristics including Gram’s stain, catalase, and oxidase test of all the 32 isolates.

Sl. No	Strain	Gram’s stain	Catalase	Oxidase
1.	C1-1	+	_	_
2.	C2-1	+	_	_
3.	C2-2	+	_	_
4.	C3-1	_	+	+
5.	C3-2	_	+	+
6.	C3-3	_	+	_
7.	P1-1	+	_	_
8.	P2-1	+	_	_
9.	P3-1	_	+	_
10.	Si1-1	_	+	+
11.	Si1-2	+	_	_
12.	Si1-3	+	_	_
13.	Si1-4	_	+	_
14.	Si1-5	+	_	_
15.	Si1-6	_	+	_
16.	Si2-1	_	+	+
17.	Si2-2	_	+	_
18.	S1-1	_	+	+
19.	S1-2	+	_	_
20.	S1-3	+	_	_
21.	S1-4	+	_	_
22.	S1-5	+	_	_
23.	S2-1	+	_	_
24.	S2-2	_	_	_
25.	S3-1	+	_	_
26.	S3-2	+	_	_
27.	S3-3	+	_	_
28.	S3-4	_	+	+
29.	S3-5	_	_	_
30.	V2-1	_	+	_
31.	V2-2	+	_	_
32.	V2-3	+	_	_

### 3.4 Qualitative analysis of LA production

#### 3.4.1 Tube test

**Figure 2:**
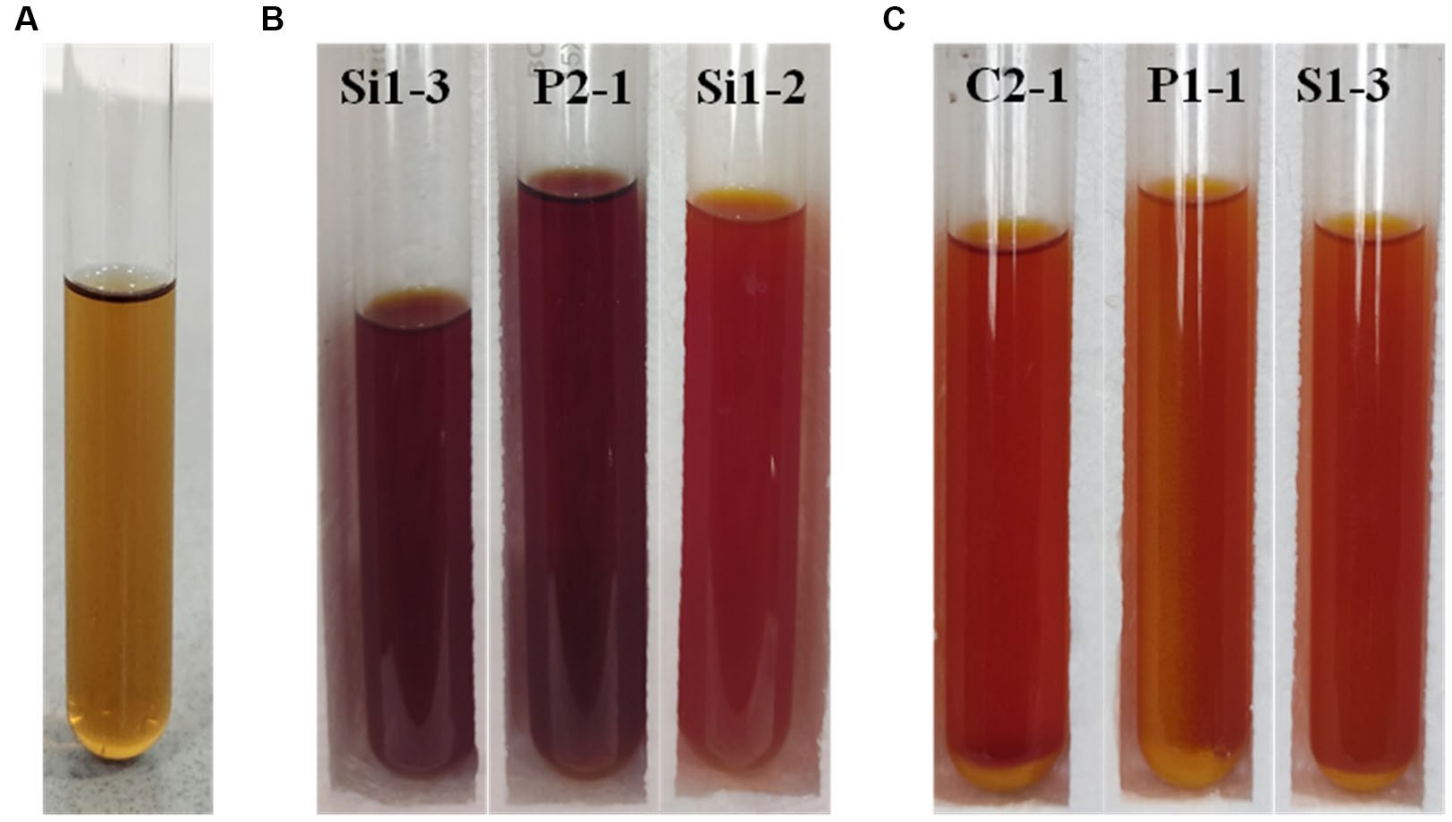
Tube test for LA production analysis **(A)** control, **(B)** 24 h incubated Si1-3, P2-1, and Si1-2, and **(C)** 48 h incubated C2-1, P1-1, and S1-3 (yellow color indicates the production of lactic acid).

The qualitative assessment of LA production from the chosen isolates was conducted using the tube test. The isolates were subjected to a 24 and 48-h investigating period to assess their ability to produce LA. The presence of a yellow color formation and acid is used as an indicator for identification purposes. Figure 2 shows the LA production exhibited by the selected isolates, as denoted by the varying intensities of yellow color. Figure 2A illustrates the levels of lactic acid production observed in the control sample. The results of the LA production are tabulated in Table 3. Based on the findings, it has been determined that all samples with the exception of S1-4, S1-5, S2-1, and V2-2 samples exhibited positive outcomes in terms of lactic acid production. Consequently, these selected samples have been chosen for subsequent investigations.

**Table 3:** Level of LA production at 24 and 48-h incubation period from tube test.

Sl. no.	Isolate	24 h	48 h
1.	C1-1	++	+++
2.	C2-1	++	+++
3.	C2-2	+	++
4.	P1-1	++	+++
5.	P2-1	+	++
6.	Si1-2	++	+++
7.	Si1-3	+	++
8.	Si1-5	++	+++
9.	S1-2	++	+++
10.	S1-3	++	+++
11.	S1-4	−	−
12.	S1-5	−	−
13.	S2-1	−	−
14.	S3-1	++	+++
15.	S3-2	++	+++
16.	S3-3	++	+++
17.	V2-2	−	−
18.	V2-3	++	+++


+, slight acid production and yellow color. ++, good acid production and yellow color. +++, increased acid production and yellow color. −, no acid production and yellow color.

#### 3.4.2 Organic acid production

**Figure 3:**
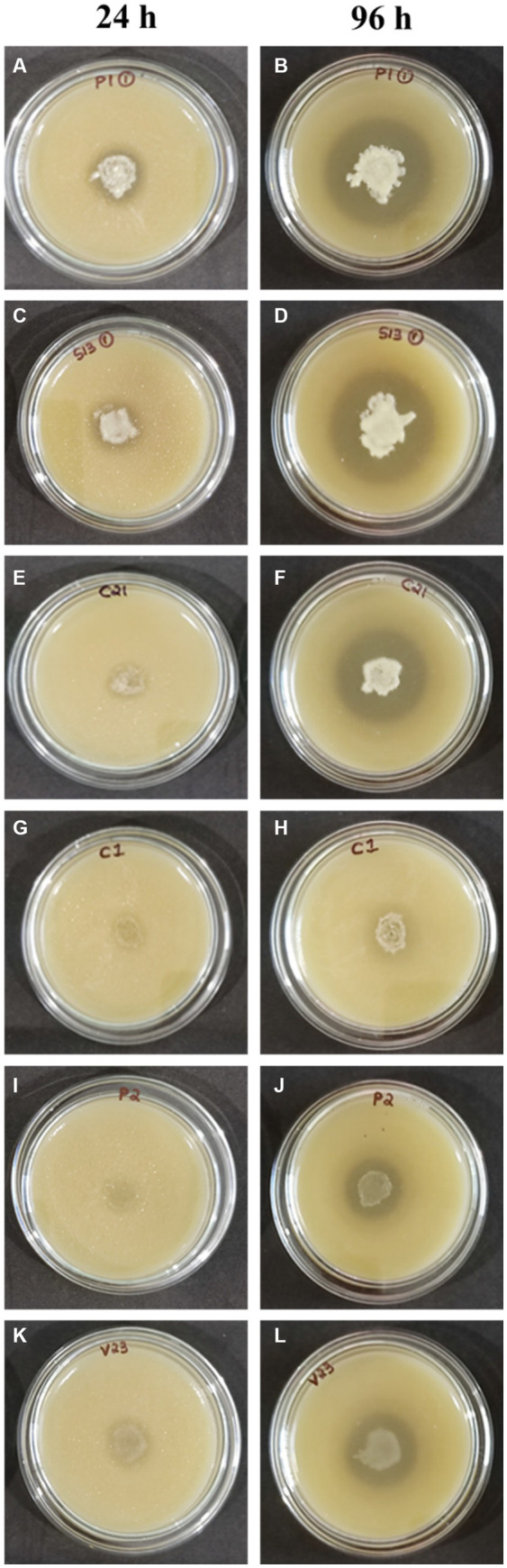
Test for organic acid production **(A,B)** P1-1, **(C,D)** S1-3, **(E,F)** C2-1, **(G,H)** C1-1, **(I,J)** P2-1, and **(K,L)** V2-3.

**Figure 4:**
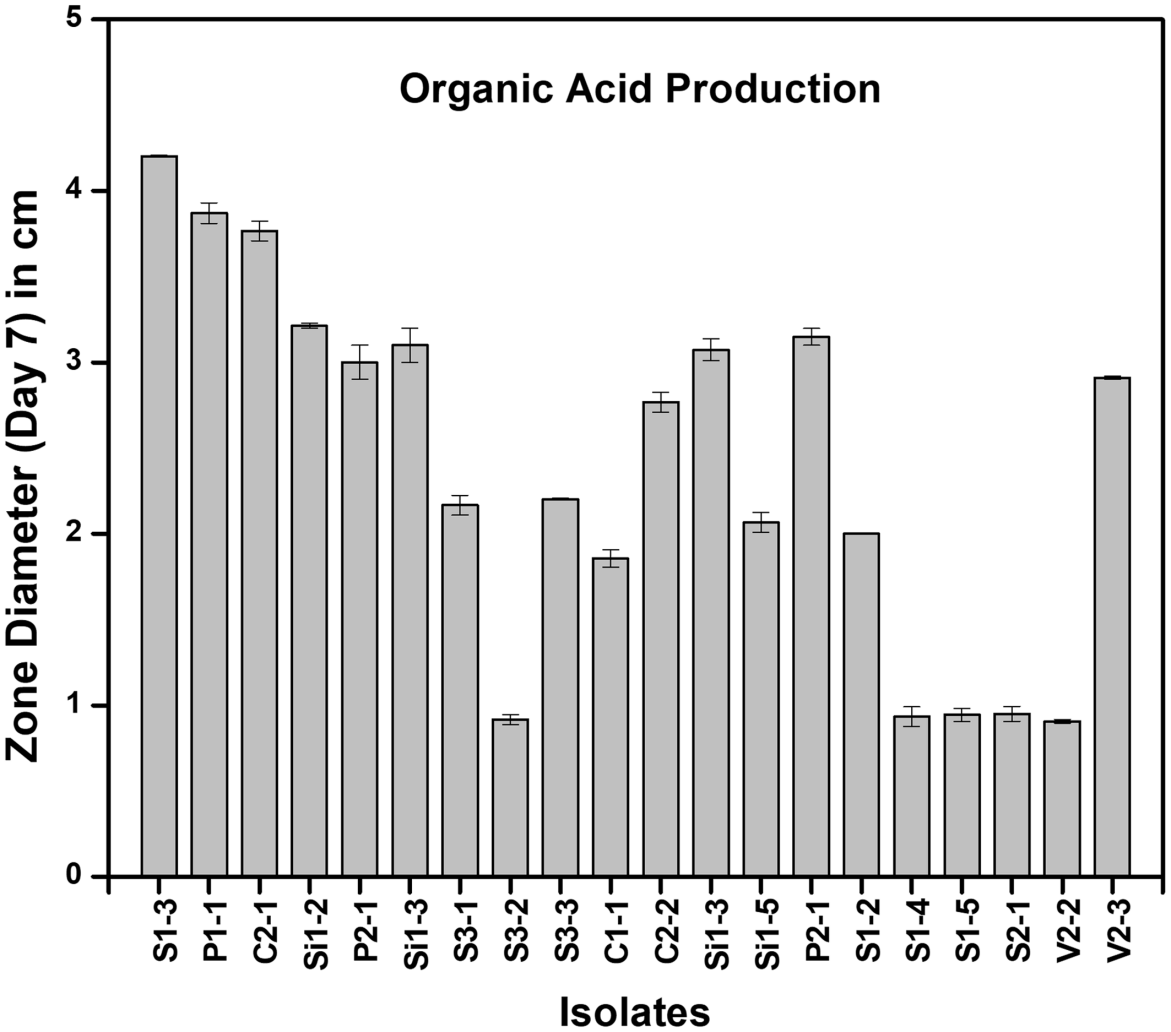
Zone of clearance measured at day 7 of organic acid production in different strains.

All 18 isolates that were determined to be LAB were introduced onto MRS agar plates containing 1% calcium carbonate. The plates were then incubated for a period of 168 h. The plates were observed at 24-h intervals to detect any zone of inhibition surrounding the inoculum. The zone diameters were measured for all 18 isolates until day 7, and plates at 24 and 96 h are shown in Figure 3. The production of organic acid by all 18 strains (day 7) has been represented in Figure 4. The initial size of the inoculum was approximately 0.8 cm. The zone diameters of all strains exhibited a progressive augmentation from the first day to the seventh day. After a 24-h incubation period, it was observed that there existed a small area of clearance surrounding the inoculum in all 18 plates. Samples S1-3 exhibited the largest zone size, measuring 3.1 cm, while sample P1-1 displayed a slightly smaller zone diameter of 3.0 cm. C2-1 and S3-2 exhibited similar values, both measuring 2.5 cm. After an incubation period of 96 h, the zone diameters of all other strains were relatively smaller. Specifically, the V2-3 strain had a diameter of 2.0 cm, the P2-1 strain had a diameter of 1.8 cm, and both the S3-1 and S3-3 strains had a diameter of 1.7 cm. After 7 days of incubation, S1-3 had a zone diameter of 4.2 cm which was the largest, followed by P1-1 and C2-1 with 3.9 cm and 3.8 cm, respectively.

#### 3.4.3 Screening for homofermentative LAB

**Figure 5:**
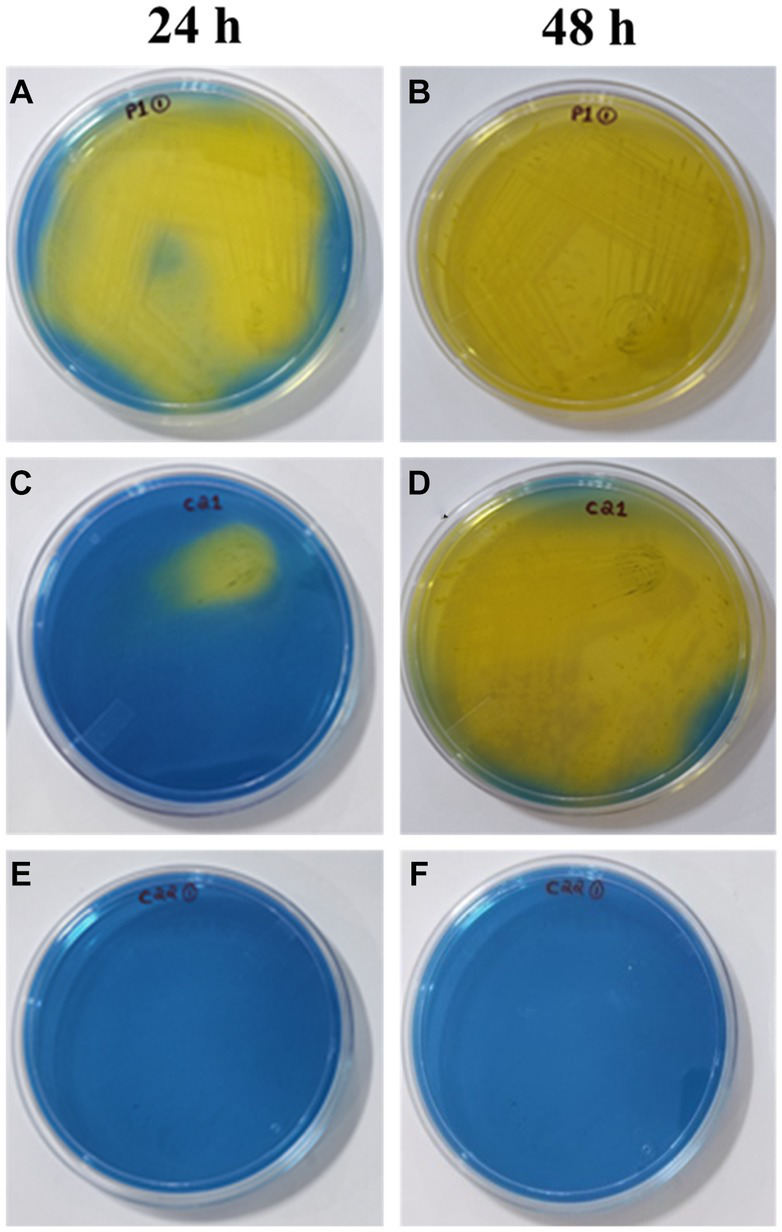
Screening of homofermentative LAB in HHD media at 24h: **(A)** (P1-1), **(C)** (C2-1), **(E)** (C2-2); 48h: **(B)** (P1-1), **(D)** (C2-1), **(F)** (C2-2). The color change from blue to yellow indicates positive result.

Heterofermentative lactic acid bacteria produce various compounds such as lactic acid, acetic acid, carbon dioxide, mannitol, and ethanol. On the other hand, homofermentative lactic acid bacteria only produce lactic acid. Homofermentative lactic acid bacteria convert fructose into lactic acid, leading to the formation of yellow color. As heterofermentative LAB exhibit lower levels of acidification, the indicator present in the growth medium may lead to variations in color formation. A total of 18 strains were analyzed for the screening of homofermentative LAB. These strains were subjected to inoculation on HHD media and followed by observation of color change at both 24 and 48 h time points (Figure 5). A noticeable transition in the color of the media, specifically from blue to yellow, was observed in the following plates: P1-1, S1-3, C1-1, S3-2, C2-1, V2-3, S3-1, P2-1, and S3-3. This change occurred consistently throughout the entire experimental period of 24 h, starting from the initial quadrant and progressing to the final streak series. In experimental samples P1-1, S1-3, and C1-1, it is observed that a significant portion of the plate underwent a transition to a yellow color within a 24-h period. Furthermore, a gradual intensification of color is noted after 48 h. In contrast, the specimens labeled as S3-2, C2-1, V2-3, S3-1, P2-1, and S3-3 exhibited a visible alteration in color in close proximity to the initial inoculation site within the first quadrant, which subsequently intensified over a period of 48 h. After 24 and 48 h, strains C2-2, Si1-2, Si1-3, Si1-5, P1-3, S1-3, S3-2, and S1-2 exhibited no observable color change and remained blue. The plates that exhibited a noticeable alteration in color were determined to be homofermentative, while the remaining isolates were identified as heterofermentative.

### 3.5 Quantitative analysis of LA production

#### 3.5.1 pH test

In the absence of pH control during the isolation of LAB, the decline in pH can be regarded as related to the degree of LA synthesis. Among the LAB strains that were found to produce lactic acid and were homofermentative, the strains P1-1, S1-3, C2-1, V2-3, P2-1, and C1-1 were used for this specified test. The results (average values along with standard deviation) of the acidifying test at 37°C using an MRS medium are tabulated in Table 4. It has been found that there is no change in pH for the control tube, which contains only MRS broth. However, all the strains used in this study exhibited a reduction in pH value from 0 h till 48 h and a slight increase in pH thereafter. The pattern of change in pH (∆pH) was more or less similar for all six isolates, with the values ranging from 2.16 to 2.19. The lowest value was recorded for all the strains at 48 h which was 3.98 for S1-3, 3.99 for P1-1, 4.01 for C2-1, 4.00 for P2-1, 4.01 for V2-3, and 4.02 for C1-1. The lowest initial pH value was observed for S1-3 which was 6.16, followed by P1-1 and V2-3 with a pH value of 6.17 and 6.18, respectively. The low pH values for all the strains as compared to the control clearly indicate the presence of LA (Hoque et al., 2010), and detailed growth kinetic studies might help in better understanding the growth pattern and time at which LA production starts exactly (Ślizewska and Chlebicz-Wójcik, 2020).

**Table 4:** pH values of media for different strains at different time points.

Strain	pH value after time t				∆pH
	*t* = 0 h	*t* = 24 h	*t* = 48 h	*t* = 72 h	
Control	6.50 ± 0	6.50 ± 0	6.50 ± 0	6.50 ± 0	0
P1-1	6.17 ± 0.006	4.09 ± 0.006	3.99 ± 0.006	4.01 ± 0	2.16
S1-3	6.16 ± 0.012	4.08 ± 0	3.98 ± 0.006	4.00 ± 0.006	2.16
C2-1	6.20 ± 0.006	4.11 ± 0.006	4.01 ± 0.006	4.01 ± 0.006	2.19
V2-3	6.18 ± 0.005	4.11 ± 0.006	4.01 ± 0.004	4.01 ± 0.006	2.17
P2-1	6.19 ± 0.01	4.07 ± 0.058	4.00 ± 0.006	4.01 ± 0.006	2.18
C1-1	6.21 ± 0.005	4.14 ± 0.012	4.02 ± 0.006	4.02 ± 0.006	2.19

#### 3.5.2 Titrimetric assay of lactic acid

The titrimetric assay was used to determine the percentage of crude LA (% purity) present in each sample (P1-1, S1-3, C2-1, V2-3, P2-1, C1-1). The samples were titrated against 0.1 N NaOH, and the titer values from three sets of titrations for the samples were 3.9 for P1-1, 3.7 for S1-3, 3.5 for C2-1, 3.1 for V2-3, 2.8 for P2-1, and 2.9 for C1-1. The percentage of lactic acid per 100 mL of sample was determined with the concordant titer values, yielding 35.11% ±0.05, 33.31% ±0.02, 31.77% ±0.017, 28.15% ±0.015, 25.41% ±0.01, and 26.32% ±0.01 for P1-1, S1-3, C2-1, V2-3, P2-1, and C1-1, respectively. The research findings indicate that the P1-1 strain exhibited the highest level of crude LA, and the lowest was C1-1. Since P1-1, S1-3, and C2-1 recorded significantly higher %LA, these three strains were selected for further analysis.

### 3.6 Thin-layer chromatography

Thin-layer chromatography (TLC) was performed to validate the presence of lactic acid. The lactic acid that had been partially purified was subjected to TLC analysis. The Rf values of the purified elution were determined, yielding values of 0.93 for P1-1, 0.9 for S1-3, and 0.96 for C2-1. These Rf values are comparable to the standard Rf value of LA [L-(+)-lactic acid extrapure AR, 88%, SRL] which was 0.97.

### 3.7 Estimation of L (+)-lactic acid

The assay was specific for L (+)-lactic acid. From the results, LA produced was 123 g/L ± 0.3, 85 g/L ± 0.2, and 62 g/L ± 0.4 for strains S1-3, P1-1, and C2-1, respectively. From the results, S1-3 recorded the highest LA production and proved to be a suitable candidate for further studies.

### 3.8 Molecular characterization

**Figure 6:**
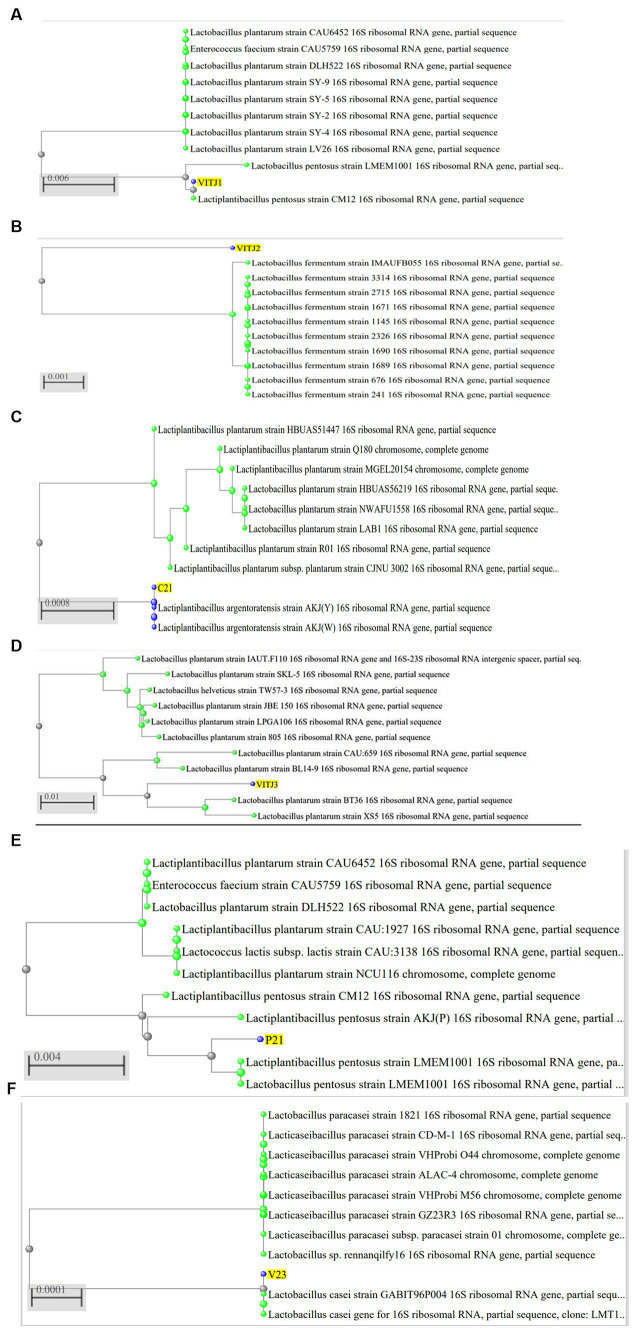
Phylogenetic analysis of 16S rDNA sequences of isolated bacteria **(A)** P1-1 (VITJ1), **(B)** S1-3 (VITJ2), **(C)** C2-1 (VITJ3), **(D)** VITJ4 (C1-1), **(E)** VITJ5 (P2-1), and **(F)** VITJ6 (V2-3).

The utilization of the BLAST database for the examination of 16S rDNA sequences aims to identify the taxonomic classification of the bacterial strain group that has been isolated. The analysis of cluster alignments revealed that the 16S rDNA sequences of the majority of the strains exhibited similarity to *Lactobacillus* group. The strain P1-1 has been classified as *Lactiplantibacillus pentosus* strain exhibiting a similarity of 99.93%. Similarly, the strain S1-3 has been identified as *Limosilactobacillus fermentum* strain with a similarity of 98.78%. Furthermore, the strain C2-1 has been recognized as *Lactobacillus argentoratensis* strain, demonstrating a similarity index of 99.92%. Strains C1-1 and P2-1 were identified as *Lactiplantibacillus pentosus* with a similarity index of 96.39 and 99.59%, respectively. V2-3 was recognized as *Lacticaseibacillus casei* with 99.81% similarity. The isolates, organism names, GenBank Accession numbers, and % similarity have been given (Table 5) along with corresponding phylogenetic trees (Figure 6).

**Table 5:** Characterization of bacterial species isolated from different organic sources.

Isolate	Organism name	Accession no.	% match
VITJ1 (P1-1)	*Lactiplantibacillus pentosus* strain CM12	OQ600641	99.93%
VITJ2 (S1-3)	*Limosilactobacillus fermentum* strain IMAUFB055	OQ733266	98.78%
VITJ3 (C2-1)	*Lactobacillus argentoratensis* strain AKJ(Y)	OQ594882	99.92%
VITJ4 (C1-1)	*Lactiplantibacillus pentosus* strain CM12	OQ600645	96.39%
VITJ5 (P2-1)	*Lactiplantibacillus pentosus* strain LMEM1001	OQ600703	99.59%
VITJ6 (V2-3)	*Lacticaseibacillus casei* strain GABIT96P004	OQ733268	99.81%

## 4 Discussion

Bacteria and certain fungi, which are frequently used to produce organic acids, especially lactic acid, have been isolated from a variety of sources. This study reports the isolation of 32 LAB strains from six different sources (organic matter-rich soil, curd, vermicompost, commercial probiotics, farmyard manure, and silage) and identified five potential homofermentative strains which were significant lactic acid producers. Our findings are consistent with the general trend observed in previous studies on the isolation of LAB from different sources; Sulmiyati et al. have reported the isolation and identification of LAB from Indonesian commercial kefir grains (Sulmiyati et al., 2018). A total of 17 *Lactobacillus* isolates were screened in a similar study conducted by Ekundayo et al. from the rhizosphere soils of guava, banana, and mango trees, gills and gastro-intestinal tracts of fish, fish-pond sediments and water, and ogi (Ekundayo, 2014). As noted by Chen et al., the isolation of distinct species of LAB from various sources may be connected to the varying nutritional conditions of the sites of isolation (Chen et al., 2005). In connection with the recent study by Satpathy et al., the current study warrants further investigation into novel sources for *Lactobacilli* isolation, including vermicompost. Similar to the findings of Bansal et al. and Shirisha et al., our results also demonstrated and confirmed the presence of different *Lactobacillus* species from conventional sources such as natural, fermented foods and probiotics (Bansal et al., 2013). As a possible source for LAB isolation, mulberry silage yielded a total of 50 isolates in the study conducted by Shokryazdan et al. (2015). A related study using raw silage samples was done by Peng et al. where they identified 49 LAB isolates (Peng et al., 2021). A recent scientific investigation conducted by Ślizewska and Chlebicz-Wójcik revealed evidence of LAB within the digestive systems of monogastric creatures, including pigs and broiler chickens, as well as in plant silage (Ślizewska and Chlebicz-Wójcik, 2020). The aforementioned reports are related to the findings of the present investigation and emphasize the significance of further investigation to identify novel sources of LAB isolation.

MRS medium is a popular growth medium for cultivating (LAB), including *Lactobacillus, Streptococcus, Pediococcus, and Leuconostoc* (Sulmiyati et al., 2018; Hayek et al., 2019). It provides nitrogenous and carbonaceous compounds, long-chain amino acids, and vital growth factors, making it an effective selective medium for LAB isolation. The study conducted by Vlková et al. employed MRS agar, M-17 agar, and Slanetz–Bartley medium to isolate *Lactobacilli, Pediococci*, and *Enterococci* from lucerne silage, all of them being LAB (Vlková et al., 2012). Despite being commonly used for LAB isolation, MRS media should be carefully considered due to its limitations, such as its lack of selectivity in differentiating between various *Lactobacilli* species. Successful isolation of pure cultures using MRS media can be challenging in the case of complex samples with mixed microbial environments. In such cases, additional selective and differential media may be required to address these limitations for more precise LAB isolation.

The findings derived from the analysis of morphological and biochemical characteristics revealed a wide range of colonies which are predominantly round in shape varying from small to large size. The majority of isolates exhibited various hues of white and yellow, including bright white, dull white, creamy yellow, or creamy white. However, few colonies displayed unique shapes and color patterns (Kim et al., 2018). A total of 18 isolates were identified as LAB based on their observed biochemical characteristics—Gram-positive, catalase-negative, and oxidase-negative (Cai et al., 2012). The results of biochemical tests for this study were similar to the results by Sobrun et al. where they have morphologically identified 17 *Lactobacilli* strains that were Gram-positive and catalase-negative (Sobrun et al., 2012).

From the results of the tube test, all other samples exhibited a favorable outcome in relation to the generation of LA with the exception of S1-4, S1-5, S2-1, and V2-2. The current finding is of significant interest as it indicates that certain microbial isolates exhibit enhanced capability of LA production under favorable conditions (Rezvani et al., 2017). The findings of the screening for organic acid production study indicate that all the 18 isolates demonstrated a zone of clearance, when cultivated on MRS agar plates supplemented with 1% calcium carbonate. The strains S1-3 and P1-1 exhibited the largest clear zones as measured. There was a gradual increase in the diameter of the zones observed from the initial day to the final day of incubation for all the strains. The findings of this study are consistent with those of Putra et al., who found that, when cultivated in MRS agar containing 0.5% CaCO_3_, LAB isolates were able to generate clear zones (Putra et al., 2018). Amarantini et al. report that the formation of organic acids, such as propionic acid, lactic acid, and/or acetic acid, dilutes the CaCO_3_ in the medium, allowing the organic acids to react with the CaCO_3_ to generate Ca lactate (Amarantini et al., 2019). According to Hoque et al., there would be a rise in the synthesis of organic acids with an increase in the incubation period (Hoque et al., 2010). These findings coincide with the current investigation.

As this study was focused on homofermentative strains, primary screening for this particular group was done using the differential media—HHD. Based on the findings presented in the experimental results for screening, it was observed that out of the 18 strains that were screened, a total of 9 strains (P1-1, S1-3, C1-1, S3-2, C2-1, V2-3, S3-1, P2-1, S3-3) were classified as homofermentative as they exhibited a greater and more extensive color change (yellow), which was observed to extend further from the site of inoculation with increasing time duration of 24–48 h. Upon careful examination of the plates that experienced this color transition, as well as those that retained a blue hue, several disparities emerge that serve to underscore the contrasting characteristics between the two categories of fermentative LAB. The heterofermentative strains displayed no or negligible color transition at their inoculation site suggesting no or reduced rate of LA production. Hence, the observable alteration in the color of the medium serves as a valuable initial indicator for clearly distinguishing the homofermentative strains (Gezginc et al., 2015).

The pH is an essential element that can significantly impact the growth and viability of bacteria (Ślizewska and Chlebicz-Wójcik, 2020). According to the results of the pH test, the media’s pH significantly decreased as the inoculum’s growth proceeded. The strains in MRS broth showed a fall in pH levels from 0 to 24 h and from 24 to 48 h, which amply demonstrates that a substantial quantity of LA generation occurs during fermentation and is inversely proportional to pH. As the data suggest, all strains had a relationship between rising LA output and falling pH levels. After a 48-h period, the pH drop tended to plateau and stayed comparatively stable. According to the research conducted by Hoque et al., various *Lactobacillus* isolates exhibit an inverse relationship between acid production and medium pH, where an increase in the former leads to a decrease in the latter, which was proved in this study as well. Based on their regional variance, *Lactobacilli* produce organic acids with a slight difference, according to this analysis (Hoque et al., 2010). The present study’s outcomes also demonstrate that all of the selected strains have the capacity to endure and thrive in a significantly low pH range of 3.8–4.0, which qualifies them as viable options for industrial uses including food preservation and the production of high-quality silage (Bao et al., 2023).

The titrimetric assay was performed to quantify crude LA produced during bacterial fermentation. The results obtained from the titrimetric assay indicate that the P1-1 strain exhibited the highest LA production per 100 mL of sample, in comparison with all other strains, with a % LA of 35.15%. Using a similar titrimetric method, Sobrun et al. determined the percentage of LA generated by the mutant LAB strains in their investigation and reported a maximum production of 21.7% LA by the highest yielding UV-generated mutant (U2) using 55% sucrose (Sobrun et al., 2012). The results of TLC serve to confirm the presence of LA in the strain cultures. The Rf values of all three strains were comparable to the standard Rf value of LA as reported in a previous study by Mohanty et al. (2015). Lee et al. have developed a TLC method to analyze short-chain fatty acids in bacterial culture broths. Thus, TLC would seem to be a very helpful tool for the presumptive identification of *Lactobacillus* strains at the genus level (Lee et al., 2001). A TLC study on enantiomeric separation of LA has been reported by Sajewicz et al. (2008). The K-LATE assay is an easy-to-use and efficient technique for precise estimation of LA. From the results using K-LATE, all the *Lactobacilli* strains proved to be efficient producers of L (+)-lactic acid, with S1-3 exhibiting the highest production. Moon et al. discovered a novel bacterium (*Lactobacillus paracasei* subsp*. paracasei* CHB2121), which produced 94.8 g/L of L(+)-lactic acid, which was examined using a K-LATE test kit (Moon et al., 2012). Iria et al. employed K-LATE enzymatic assay kits and biosensors to evaluate the lactate composition in various food specimens and reported a mean concentration of 5.7 g/L ± 0.4 L(+)-lactic acid in yoghurt samples (Bravo et al., 2017). *Lb delbrueckii* strain was found to produce 107 and 120 g/L of D (−)-lactic acid from sugarcane molasses and sugarcane juice, respectively, used as fermentation substrate, in a study by Calabia and Tokiwa (2007). The LA production capability of S1-3 seems to outperform all the strains mentioned above, and thus, it serves to be a promising candidate for commercial LA production after further studies.

The BLAST database effectively facilitated the determination of the taxonomic classification of the bacterial strains that were isolated and identified to be potential homofermenters. The strains were identified as *Lactiplantibacillus pentosus* (P1-1), *Limosilactobacillus fermentum* (S1-3), *Lactiplantibacillus argentoratensis* (C2-1), *Lactiplantibacillus pentosus* (C1-1), *Lacticaseibacillus casei* (V2-3), and *Lactiplantibacillus pentosus* (P2-1). The strains used to produce lactic acid industrially have recently become almost exclusively proprietary, and the majority of the LAB used are thought to be members of the genus *Lactobacillus* (Meenambigai and Nair, 2021). These findings demonstrate significant advantages for the production of LA on a commercial scale using wild LAB strains. Our results suggest a more nuanced understanding of novel organic sources, than that provided by previous research.

## 5 Conclusion

Six homofermentative strains of *Lactobacilli* were successfully isolated and identified from various sources of organic material, including vermicompost and sugarcane soil. Among the six, three showed exceptional production capacities which were quantitatively analyzed through Titrimetric assay, validated using TLC and amount of L (+)-lactic acid estimated using K-LATE (Megazyme assay kit). Based on the initial screening of 32 isolates and a combination of qualitative and quantitative analysis, the strain *Limosilactobacillus fermentum* (S1-3) appeared to be the most suitable for large-scale fermentation and industrial use in terms of lactic acid production. This isolate was observed to provide better results as compared with the strains reported in previous studies. In addition, the isolation of *Lacticaseibacillus casei* strain from vermicompost opens up new research avenues for homofermentative LAB isolation from as-yet-undiscovered possible organic sources in future. Hence, these strains displaying the capacity to synthesize LA possess notable prospects for incorporation into various applications, including food production, human health, agriculture, and the fabrication of biodegradable polymers like PLA.

## Data availability statement

The datasets presented in this study can be found in online repositories. The names of the repository/repositories and accession number(s) can be found in the article/supplementary material.

## Author contributions

JS: Conceptualization, Data curation, Formal analysis, Investigation, Methodology, Writing – original draft. AS: Conceptualization, Supervision, Writing – review & editing.
